# Genetic Analysis of Completely Sequenced Disease-Associated MHC Haplotypes Identifies Shuffling of Segments in Recent Human History

**DOI:** 10.1371/journal.pgen.0020009

**Published:** 2006-01-27

**Authors:** James A Traherne, Roger Horton, Anne N Roberts, Marcos M Miretti, Matthew E Hurles, C. Andrew Stewart, Jennifer L Ashurst, Alexey M Atrazhev, Penny Coggill, Sophie Palmer, Jeff Almeida, Sarah Sims, Laurens G Wilming, Jane Rogers, Pieter J. de Jong, Mary Carrington, John F Elliott, Stephen Sawcer, John A Todd, John Trowsdale, Stephan Beck

**Affiliations:** 1 Department of Pathology, Immunology Division, University of Cambridge, Cambridge, United Kingdom; 2 Wellcome Trust Sanger Institute, Genome Campus, Hinxton, Cambridge, United Kingdom; 3 Juvenile Diabetes Research Foundation/Wellcome Trust Diabetes and Inflammation Laboratory, Cambridge Institute for Medical Research, University of Cambridge, Wellcome Trust/MRC Building, Addenbrooke's Hospital, Cambridge, United Kingdom; 4 Alberta Diabetes Institute (ADI), Department of Medical Microbiology and Immunology, Division of Dermatology and Cutaneous Sciences, University of Alberta, Edmonton, Canada; 5 Children's Hospital Oakland Research Institute, Oakland, California, United States of America; 6 Basic Research Program, SAIC-Frederick, Inc., Laboratory of Genomic Diversity, National Cancer Institute, Frederick, Maryland, United States of America; 7 Department of Clinical Neurosciences, University of Cambridge, Addenbrooke's Hospital, Cambridge, United Kingdom; The Jackson Laboratory, United States of America

## Abstract

The major histocompatibility complex (MHC) is recognised as one of the most important genetic regions in relation to common human disease. Advancement in identification of MHC genes that confer susceptibility to disease requires greater knowledge of sequence variation across the complex. Highly duplicated and polymorphic regions of the human genome such as the MHC are, however, somewhat refractory to some whole-genome analysis methods. To address this issue, we are employing a bacterial artificial chromosome (BAC) cloning strategy to sequence entire MHC haplotypes from consanguineous cell lines as part of the MHC Haplotype Project. Here we present 4.25 Mb of the human haplotype QBL (HLA-A26-B18-Cw5-DR3-DQ2) and compare it with the MHC reference haplotype and with a second haplotype, COX (HLA-A1-B8-Cw7-DR3-DQ2), that shares the same *HLA-DRB1, -DQA1,* and *-DQB1* alleles. We have defined the complete gene, splice variant, and sequence variation contents of all three haplotypes, comprising over 259 annotated loci and over 20,000 single nucleotide polymorphisms (SNPs). Certain coding sequences vary significantly between different haplotypes, making them candidates for functional and disease-association studies. Analysis of the two DR3 haplotypes allowed delineation of the shared sequence between two HLA class II–related haplotypes differing in disease associations and the identification of at least one of the sites that mediated the original recombination event. The levels of variation across the MHC were similar to those seen for other HLA-disparate haplotypes, except for a 158-kb segment that contained the *HLA-DRB1, -DQA1,* and *-DQB1* genes and showed very limited polymorphism compatible with identity-by-descent and relatively recent common ancestry (<3,400 generations). These results indicate that the differential disease associations of these two DR3 haplotypes are due to sequence variation outside this central 158-kb segment, and that shuffling of ancestral blocks via recombination is a potential mechanism whereby certain DR–DQ allelic combinations, which presumably have favoured immunological functions, can spread across haplotypes and populations.

## Synopsis

A group of genes involved in the human immune system are contained within a surprisingly short section of Chromosome 6 that has long been recognised as the most important genomic region in relation to disease susceptibility. Discerning the actual genes playing a role in disease has proved difficult mainly because the region contains numerous genes and is also the most genetically variable in the genome. Within this jungle of variation, the research reported here has identified and characterised a discrete segment shared by two individuals that is virtually devoid of variation—a polymorphism desert. The conservation of this segment amongst a background of extreme variation suggests both an ancient origin and genetic exchange in early human history. These observations are important in evolutionary terms as they reveal a potential mechanism whereby certain genetic segments associated with favourable immune functions have spread across human populations. Within medical terms this may also explain contrasting disease risks in people from different ethnic backgrounds. Public access to these data will help researchers find specific variants conferring disease susceptibility or resistance and, as in this report, rule out regions for conveying specificity to certain diseases.

## Introduction

The classical major histocompatibility complex (MHC) containing the human leukocyte antigen (HLA) loci on human Chromosome 6p21.31 is a gene-dense region spanning nearly 4 Mb. The region plays an important role in disease resistance and susceptibility. About 30% of the approximately 150 constituent-expressed genes encode functioning immune-related molecules. The allelic and genetic structure of the MHC is complex. It harbours some of the most polymorphic genes in the genome, and sequences differ in size and gene composition partly as a result of non-allelic homologous recombination [1]. These extreme levels of polymorphism and dense genetic organisation, including highly reiterated sequences, have made particular parts of this biologically and medically important region less accessible to genome-wide analyses such as those employed by the International SNP and HapMap projects (International SNP Map Working Group 2001, [http://snp.cshl.org] [2]; The International HapMap Consortium 2003, [http://www.hapmap.org] [3]). In addition, polymorphism studies within the MHC have historically focused on small areas or have been anecdotal. Consequently, knowledge of sequence variation across the complex has been fragmentary, and conventional single nucleotide polymorphism (SNP) densities have not been sufficient to provide a contiguous map of allelic variation that captures the genetic diversity of the MHC, which is a limitation in the identification of MHC variants primarily associated with disease.

The MHC Haplotype Project (http://www.sanger.ac.uk/HGP/Chr6/MHC) was designed to address this problem by cloning and sequencing of bacterial artificial chromosome (BAC) library clones derived from unrelated consanguineous cell lines homozygous for certain HLA haplotypes thereby eliminating the possibility of heterozygosity [4]. This resource will provide ready access to the genomic sequences of eight different MHC haplotypes and their resulting SNP and deletion/insertion polymorphism (DIP) variations, their ancestral relationships, and the accurate annotation of all loci and their splice variants. To date, the study has contributed over 60% of the total number of recorded SNPs (dbSNP124) across the MHC and provided more than 50% of the available SNPs for the most-recent high-resolution haplotype map of the MHC that can be applied to association studies of MHC-linked diseases [5].

In total, autoimmune diseases affect approximately 5% of the population and include type 1 diabetes, rheumatoid arthritis, and multiple sclerosis. Linkage scans and association-mapping studies have identified the MHC as influencing most, if not all, autoimmune conditions [6–12] along with certain infectious diseases, including malaria and AIDS [13,14]. For some common autoimmune diseases, the MHC provides by far the largest genetic contribution by a single chromosome region. For example, the MHC accounts for at least 30% of the familial aggregation in type 1 diabetes and rheumatoid arthritis [15–17], with additional chromosome loci outside the MHC contributing smaller individual genetic effects. The highly polymorphic HLA class I and class II loci are believed to be the major determinants of MHC-associated disease, but in general the precise MHC variants influencing these conditions remain unknown despite intense study. Hampered by inadequate knowledge of sequence variation across the complete MHC, fine-mapping of the primary causal variants has been additionally confounded by the complexity of associations in which several MHC genes may be involved. Moreover, the extensive linkage disequilibrium (LD) between certain genes of the MHC, notably *HLA-DRB1* and *-DQB1,* along with the high gene density of the region makes it difficult to rule out a contribution from other linked genes, such as those in class III and the extended class I regions.

We have previously reported the complete and contiguous sequence and annotation for two human MHC haplotypes, PGF (HLA-A3-B7-Cw7-DR15-DQ6) and COX (HLA-A1-B8-Cw7-DR3-DQ2), each spanning just less than 5 Mb [18] (see Table S1 for DNA typing profiles) and producing 16,013 SNPs. Here we describe 4.25 Mb of sequence and the variation content from a second DR3-DQ2 haplotype, QBL (HLA-A26-B18-Cw5-DR3-DQ2). Currently, these MHC haplotypes represent the only long-range single haplotype sequences in the human genome [18]. The cell lines were selected for study from the 10th International Histocompatibility Workshop panel [19]. The haplotypes chosen are disease-associated and common, with northern European frequencies on the order of 10%. The COX haplotype has been associated with susceptibility to a wide range of diseases, including type 1 diabetes, systemic lupus erythematosus, and myasthenia gravis [20]. The PGF haplotype provides protection against type 1 diabetes and predisposes to other diseases such as multiple sclerosis and systemic lupus erythematosus [21–24]. The QBL haplotype is positively associated with Graves' disease and type 1 diabetes [25].

The MHC lends itself to analysis of meiotic recombination because of the high density of variation. Comparison of two full-length DR3 haplotypes allowed demarcation of sequence shared between two HLA-related haplotypes that show differing disease associations. We assessed the LD structure of a 158-kb segment of shared sequence identified in the two DR3 haplotypes at the population level using high-density SNP-typing data for 180 Centre d'Etude du Polymorphisme Humain (CEPH) founder chromosomes. These findings emphasize the importance of fine-scale LD structure of the MHC and suggest that DR/DQ segment exchange between MHC haplotypes in relation to recombination hotspots may be responsible for contrasting disease risk related to non-DR/DQ loci and to different ethnic backgrounds.

## Results

### Sequence

**Figure 1:**
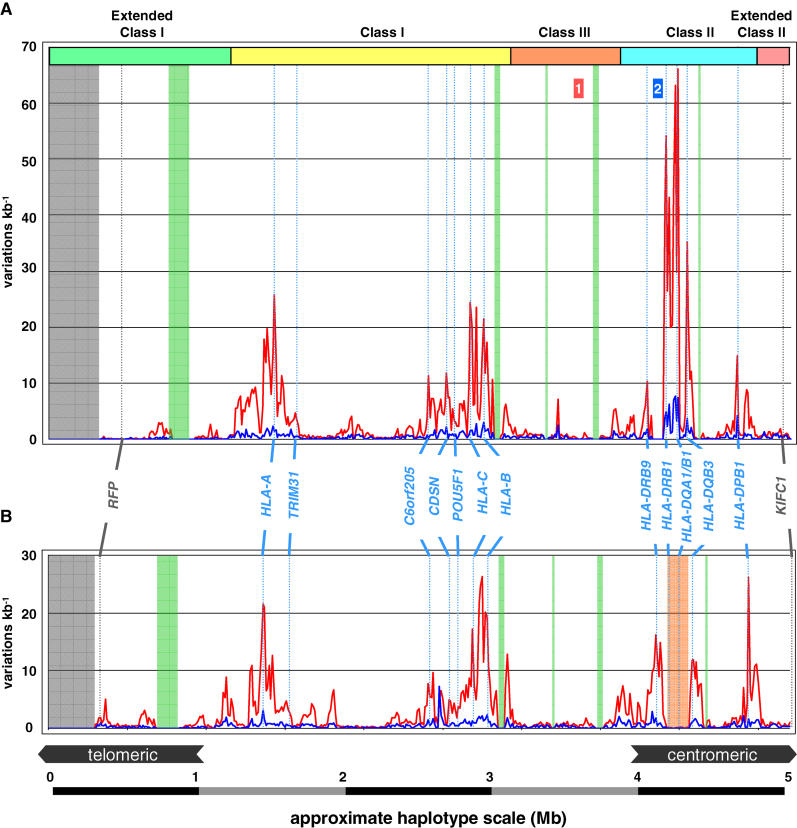
Positional Distributions of Variations between PGF and QBL and COX and QBL (A) Shows the distribution for PGF and QBL and (B) shows COX and QBL. MHC sequences were divided into 10-kb bins, and variations were calculated in each bin. Results are expressed as variations per 1 kb. Red and blue plots relate to SNP and DIP variations respectively. The sequence is interrupted by five gaps, shown as green vertical bars, where BACs encompassing these regions could not be identified from the clone library, which by comparison with PGF comprise a total of approximately 317 kb. The lengths and gene content of these gaps were as follows, from left to right: 159 kb including *OR2U1P* to *OR12D2;* 51 kb containing *HCP5;* 26 kb containing *C6orf26, C6orf27,* and the three exons of 3′ end of *MSH5;* 53 kb containing *CREBL1, FKBPL,* and six exons of the 5′ end of *TNXB;* and 27 kb containing *HLA-DOB*. These gaps do not represent large genomic deletions within the QBL haplotype since exonic sequence from selected genes within these regions were successfully amplified from QBL genomic DNA and sequenced to confirm their identity. The grey shaded area at the telomeric end of the map represents sequence for which overlap was not obtained and was therefore outside the area that was compared. Boundaries of the class I, II, and III regions are shown. The positions of *RFP* and *KIFC1* that define the ends of the MHC haplotype sequencing project are indicated. Landmark genes are labelled in blue. Regions 1 and 2 are the RCCX module and the *HLA-DRB* region, respectively. The *HLA-DRB3* and *HLA-DQB3* region, which shows little variation between COX and QBL haplotypes, is shaded in orange.

Approximately 4.25 Mb of the QBL haplotype sequence, from *RFP* to *KIFC1,* spanning the MHC class I, class II, and class III regions, along with part of the extended class I region, was obtained from large-insert BAC clones by shotgun sequencing. There were five small gaps of the size range 26–159 kb in the QBL sequence, owing to incomplete clone coverage, which by comparison to PGF comprised a total of only about 317 kb (see Figure 1). A test was conducted to ensure that QBL BACs were derived from a single haplotype by comparing adequately overlapping reads from the shotgun sequencing strategy extending into adjacent BAC clones. The QBL BAC overlaps indicated homozygosity over the entire MHC for the QBL cell line.

### Annotation and Gene Content

The human-curated annotation of the QBL haplotype sequence revealed a total of 259 loci, including 149 coding, 27 transcribed loci, and 83 pseudogenes. All these loci are the same as those found in the PGF and COX haplotypes [18], after taking into account the gaps in the QBL sequence contig, and the conformations of the DR and RCCX *(RP*-*C4A/B*-*CYP21*-*TNXB)* regions (see below).

QBL and COX do not differ in respect to their *HLA-DRB* gene composition, and both haplotypes contain a single *C4* gene, although of a different allele (see below). The intronless pseudogene, *PPP1R2P1,* has a full-length open reading frame in QBL, as found previously in PGF and COX [18], and does not possess the frameshift mutation present in the original reference MHC gene sequence [26,27]. Four other notable and potentially functional alterations to loci were observed within the class I region in an area shown to be important in susceptibility to psoriasis [28–30]: (1) The locus *C6orf205* encoding a putative transmembrane protein [31,32] possesses a coding minisatellite in exon 2: PGF and COX both possess only 27 copies, whereas QBL possesses 31 copies of the 45-mer repeat sequence, thus extending the coding sequence (CDS) by 180 nucleotides and the translated CDS by 60 amino acids. (2) In QBL, *PSORS1C1,* a psoriasis candidate locus [33], has a deletion of one nucleotide in a polyC tract in exon 5 (at base position 118 in the PGF CDS) compared to COX and PGF. This produces a frameshift in the spliced mRNA transcript and a premature stop codon in the following exon, which would shorten the CDS by 266 base pairs (bp) (resultant CDS 192: 64 amino acids; compared to CDS 459: 152 amino acids in PGF and COX), resulting in a novel stretch of 24 amino acids in the terminal end of the protein product. (3) *POU5F1* encodes a POU homeodomain–containing transcription factor. A SNP identified in PGF disrupted the Met start codon of the alternative splice isoform, resulting in a shorter open reading frame in PGF than in QBL and COX. (4) *HCG22* (HLA complex group 22) encodes a novel transcript. The second exon in the QBL haplotype is approximately 174 bp shorter than in either PGF or COX; however, as no open reading frame for this transcript could be identified, the significance of this observation is not known. Other changes observed are consistent with known allelic polymorphisms (see Table S1).

The annotation of the haplotypes is available as a general resource through the Vertebrate Genome Annotation (VEGA) database (http://vega.sanger.ac.uk) with PGF as the reference haplotype. All annotation is to standards set by the Human Annotation Workshop (HAWK), providing accuracy greater than currently possible through in silico methods [34]. The annotation shown reflects the cDNA, EST (expressed sequence tag), and protein evidence available at the time of analysis, and it is therefore appreciated that this will change with future information. Splicing cDNA or EST evidence was required for annotation of all splice variants.

### Sequence Variation

Table 1 summarises all variations observed between the PGF reference and QBL haplotypes. Across 4.25 Mb of sequence, this included a total of 17,695 variations, of which 15,345 were SNPs and the remainder (13%) were DIPs. There were 528 SNPs in coding regions. The mean SNP density was 2.12/kb within coding regions. Exonic UTR and intronic DNA displayed higher variation densities of 2.50/kb and 2.48/kb, respectively, and even higher variation densities were observed in intergenic non-repeat DNA, pseudogene sequences, and interspersed repeats (average 3.76/kb).

**Table 1:**
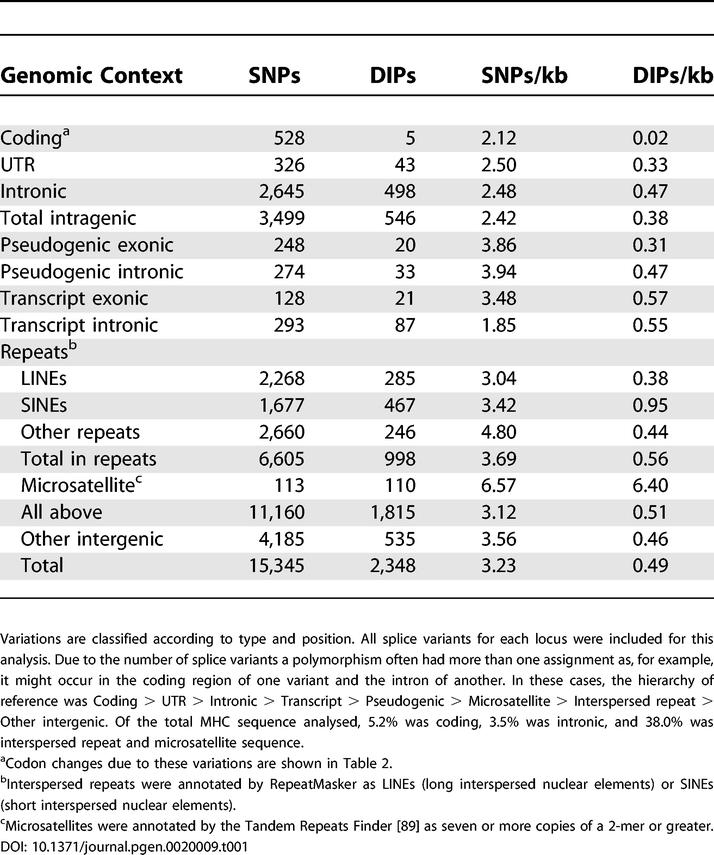
Sequence Variations between QBL and PGF

**Table 2:**
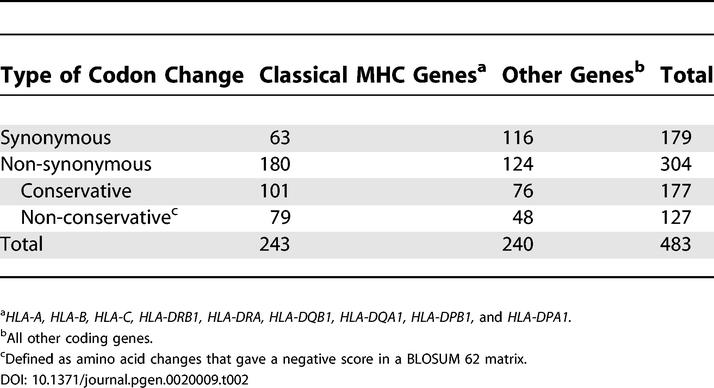
Codon Changes due to Coding SNPs between QBL and PGF

The coding SNPs between PGF and QBL were contained in 483 codons: 243 in the nine classical HLA loci and 240 in all other loci (Table 2). Categorization of the coding SNPs into types of codon changes introduced showed that the nine classical MHC genes had a non-synonymous:synonymous (N:S) ratio of approximately 3:1 (Table 2). These data are consistent with positive selection acting on these genes [35,36]. Of the non-synonymous amino acid changes, 43% were non-conservative, as defined by a negative score in a BLOSUM 62 matrix [37]. In contrast, the pooled set of non-classical MHC genes had a N:S ratio of 1:1. Amongst the non-synonymous changes, the pooled sets of both classical and non-classical MHC genes had similar proportions of non-conservative changes.

The coding DIPs observed between PGF and QBL (Table 1) altered the CDS of *MICA* and *HLA-DQA1* as is consistent with known allelic polymorphisms (see Table S1), and *C6orf205* and *PSORS1C1* as described earlier. A plot of size distribution of all DIPs within the range of 1 bp to 36 bp showed a negative correlation between frequency and DIP size with a log-linear distribution (Figure S1).

Thirty-four large DIPs (96–5,157 bp) were identified between PGF and QBL haplotypes (Table S2), some of which have previously been identified (e.g., [38–42]); 22 were found by comparison of previously reported haplotypes PGF and COX [18]. Fifteen DIPs (of the 34) resulted from *Alu* element insertions. The majority of these belonged to the younger *Alu Y* subfamily, and more specifically five were of the *Ya5* and *Yb8* types, which emerged after the divergence of humans from African apes [43]. There were also differences between PGF and QBL in the presence/absence of more ancient sequences, such as *AluS* and repeats of the HERV (human endogenous retrovirus), LINE (long interspersed nuclear element), LTR (long tandem repeat), MER (mammalian interspersed repetitive element), and SVA (SINE-VNTR-Alu) families. These differences were concentrated between DRB1 and DQB1 in the class II region but were more evenly distributed across the class I region, consistent with an early divergence of the PGF and QBL haplotypes in the DR–DQ and class I regions relative to other regions of the MHC. These large DIPs account for at least 37 kb of sequence.

For comparative study of sequence diversity, we measured heterozygosity per nucleotide site (π). The π value between any two haplotypes throughout the genome has been estimated to lie between 5 × 10^−4^ and 9 × 10^−4^ and is known to vary by chromosomal region [2,44–46]. The level of SNP variation between COX and PGF (3.4 × 10^−3^), and QBL and PGF (3.6 × 10^−3^) is 4- to 7-fold higher than estimates for genome-wide heterozygosity. In comparing π for PGF versus COX or QBL, there was bias by selection of HLA-disparate haplotypes. The π value between QBL and COX (2.7 × 10^−3^), two DR3-DQ2 haplotypes, was therefore expected to be less than between totally disparate HLA haplotypes, but it nevertheless remained significantly higher than genome-wide estimates. Approximately 40% (6,230 of 15,345) of the SNPs and 57% (1,332 of 2,350) of the DIPs from QBL were distinct from those already identified in the comparison of PGF–COX. For all haplotype comparisons, however, the relatively high level of variation observed was mostly due to the peaks surrounding the classical *HLA* loci, since sequence diversity outside these areas was comparable to the genome-wide estimates (data not shown).

To display the variations between haplotypes, we plotted variation density against genomic position (Figure 1). As expected, the highest levels concentrated in peaks overlying the classical class I and class II loci, and are usually explained by balancing selection acting on the peptide-binding domains of the *HLA* loci [35,36] with “hitch-hiking” of neighbouring mutations [47]. Some smaller peaks were sited over genes other than classical HLA loci, consistent with independent selection for variation. An example of this concentrated variation was previously observed in the comparison between PGF and COX telomeric of *HLA-C* from *CDSN* to *POU5F1* [18], and a similar peak was identified in the comparison of COX with QBL.

### *HLA-DR* Region Sequence Variation

The *HLA-DRB* region is known to be extremely polymorphic both in terms of SNPs, and of the insertion and deletion of large fragments of genomic sequence [48], such that different haplotypes have missing or alternative arrangements of *HLA-DRB* genes and pseudogenes [49]. QBL and COX possessed the same *HLA-DRB* gene composition that was distinct from PGF, with both haplotypes sharing the loci arrangement *DRB9, DRB3, DRB2,* and *DRB1*. These two haplotypes therefore carry two functional *DRB* genes *(DRB3* and *DRB1)* and two *DRB* pseudogenes *(DRB9* and *DRB2)*. The high degree of variation between PGF and COX/QBL in the DR/DQ region suggests that the HLA haplotypes embodied in PGF (DR15-DQ6) and COX/QBL (DR3-DQ2) are ancient and highly diverged relative to each other.

**Figure 2:**
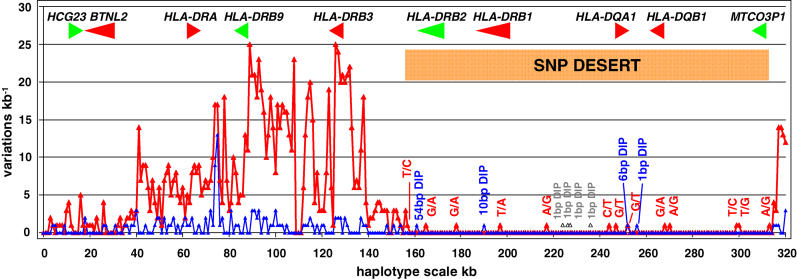
Positional Distributions of Variations between COX and QBL in the *HLA-DR* Region MHC sequences were divided into 10-kb bins, and variations were calculated in each bin. Results are expressed as variations per 1 kb. Red and blue plots relate to SNP and DIP variations respectively. Within a stretch of approximately 160 kb between *HLA-DRB3* and *HLA-DQB3,* only 14 SNPs and six small DIPs, comprising 1 bp, 6 bp, 10 bp (five copies of a dinucleotide repeat), and 54 bp (two copies of 27 mer), were contained. None of the variations located to coding sequence or the defined promoter regions of the HLA class II genes [86]. Four 1-bp DIPs, labelled in grey, were identified between *DRB1* and *DQA1* where LR-PCR products were used to close a small gap resulting from clone deficit. These DIPs were located in polyA/T tracts in which the probability of *Taq* slippage in PCR products is much higher than in in-vivo amplified plasmid DNA such that their authenticity was questionable and they were excluded from analyses (Figure S2 shows one alignment of sequence traces with differing polyT tracts).

Interestingly, when part of the *HLA-DRB* region containing the genes *HLA-DRB1, HLA-DQA1,* and *HLA-DQB1* and pseudogenes *HLA-DRB2* and *MTCO3P1* was compared between COX and QBL only 14 SNPs were found in 158 kb; approximately 1 per 10 kb (π = 8.2 × 10^−5^) (Figure 2). The coding sequences of all these genes were identical in the two haplotypes. By contrast, although this region is not entirely represented in the PGF haplotype, in the 128 kb of sequence also found in PGF, there were 3,754 SNPs when compared with QBL (π = 2.9 × 10^−2^), and 3,808 when compared with COX (π = 3.0 × 10^−2^). Using a range of plausible mutation rates from 1.3 to 2.1 × 10^−8^ substitutions per site per generation [50], based on the assumption that the human and chimpanzee lineages split 6 million years ago (and in absence of selection), we estimate that the time to the most recent common ancestor of two sequences that have accumulated only 14 SNPs within 158 kb is 2,100–3,400 generations.

**Figure 3:**
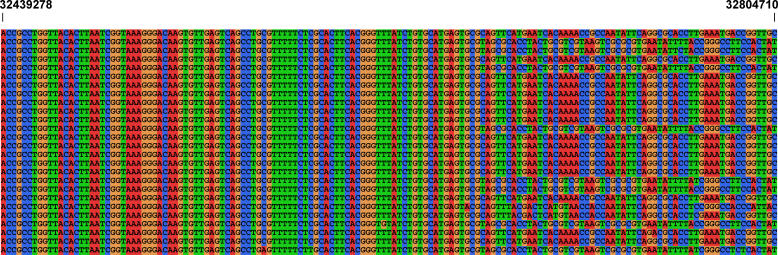
Haplotype Alignment of the Region Presenting Differing Variation Rates The alignment covers the centromeric side of the DR–DQ 158-kb DNA segment (left half, low variation) and the adjacent DNA segment (increased variation). Coordinates refer to Chromosome 6 build NCBI35. Rows represent the allelic state for 26 single chromosomes with the same DRB1*1501-DQA1*0102-DQB1*0602 (DR15–DQ6) haplotype at successive SNPs which are represented by columns (A, red; C, blue; G, orange; and T, green). Identity is interrupted at a position perfectly matching with a recombination hotspot coordinate [5,53] represented as hotspot number 2 in Figure 4.

The presence of the SNP desert and its boundaries was evaluated at the population level by assessing the diversity in the DR–DQ region in chromosomes sharing the DRB1*0301-DQA1*0501-DQB1*0201 (DR3-DQ2) haplotype common to COX and QBL. This analysis was carried out using high-density SNP-typing data for 140 phased CEPH founder chromosomes [5] with available HLA-typing information. For the 158-kb segment defined above, the mean sequence pairwise differences considering 12 chromosomes sharing the DR3-DQ2 segment is ten times lower than the divergence estimate based on the adjacent centromeric 158-kb fragment. We then investigated the diversity in these two regions on 26 chromosomes sharing the most common HLA class II haplotype in this sample: DRB1*1501-DQA1*0102-DQB1*0602 (DR15–DQ6). In this case, the divergence estimate for the DR–DQ region is 200 times lower when compared to the 158-kb segment immediately centromeric (Table 3). High identity can be predicted when SNP typing is carried out in a sub-sample of chromosomes bearing the same DRB1*1501–DQB1*0602 (DR15–DQ6) haplotype. Nevertheless, the abrupt transition from almost complete identity to increased diversity in fewer than 5 kb on the centromeric side of the *DR–DQ* 158-kb segment is remarkable. The boundary between these adjacent segments of contrasting diversity levels can be observed in the alignment of multiple haplotypes sharing DRB1*1501–DQB1*0602 (DR15–DQ6) (Figure 3). This 5-kb region separating these two DNA segments corresponds to a recombination hotspot identified by sperm-based genotyping [51,52]. As expected, the variation level was higher at more distant loci such as HLA-A and HLA-B-HLA-C (Table 3).

**Table 3:**
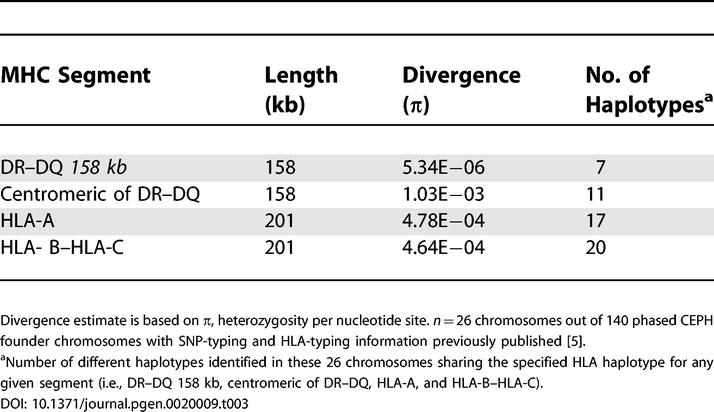
Divergence Estimates for Different Regions in the MHC Observed on 26 Chromosomes Sharing the DRB1*1501-DQA1*0102-DQB1*0602 (DR15–DQ6) Haplotype

**Figure 4:**
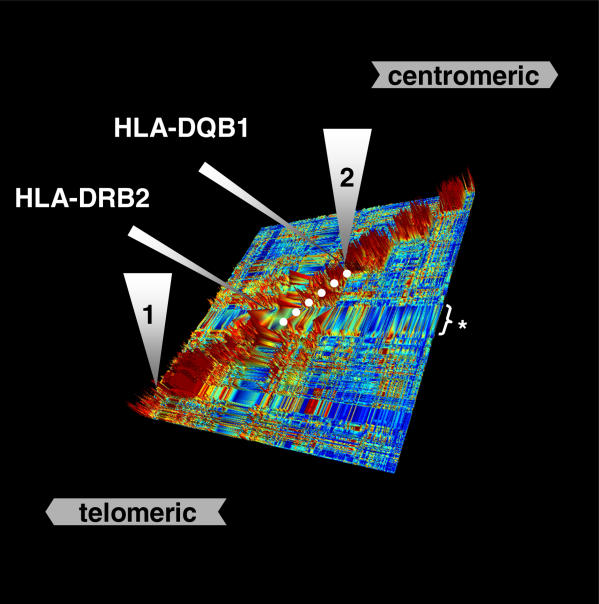
LD Structure around the *HLA-DR* Region High-resolution view of the *HLA-DR* region, as represented by GOLDsurfer three-dimensional view of D′ values [81]. The position of the 158-kb segment shared by identical by descent between COX and QBL is shown by a dashed white line. High LD areas (red blocks) are separated by LD breaks. The first LD break (1) corresponds to a recombination hotspot mapped between *NOTCH4* and *C6orf10* in the class II–III boundary region. Another LD break (2) is visualized at another recombination hotspot centromeric of *HLA-DQB1* at the boundary of the SNP desert between COX and QBL. This is followed centromerically by a further four LD breaks corresponding to recombination hotspots mapped at *BRD2*/*HLA-DOA* interval, within *HLA-DMB,* within *TAP2* and *HLA-DQB2*/-*DOB* interval [5,51,53]. An asterisk (*) indicates a region of depleted SNP data, likely owing to substantial genotyping failure in an area with an extreme level of polymorphism.

In order to investigate potential analogies at different HLA loci, we analysed pairwise variation levels at the *HLA-C-B* region and at its adjacent centromeric segment in a sample of 22 chromosomes sharing the HLA-C*0702–HLA-B*0702 haplotype, the most common *HLA-C-B* haplotype in the sample. For the 201-kb segment containing the *HLA-C-B* genes, the mean number of pairwise differences between all pairs of haplotypes (5.39 × 10^−6^) was ten times lower than the value obtained for the adjacent 201-kb segment on its centromeric side (6.39 × 10^−5^). Comparable variation levels have been observed in both *HLA-DRB-DQ* and *HLA-C-B* blocks when strengthening homozygosity in each region by sub-sampling only chromosomes sharing the most frequent HLA haplotype. However, variation level in the 158-kb segment immediately centromeric to the *DR–DQ* block was more prominent. Also, the *HLA-C-B* block boundary was not as steep and narrow as in the class II block.

The 158-kb sequence, which is clearly identical by descent between COX and QBL, is abruptly interrupted at coordinate AL731683.12 73150 on the centromeric side in the COX sequence contig beyond which the SNP density rises to >1/kb. Therefore, we examined the LD profile in order to investigate the distribution of LD patterns in the region and search for potential LD breaks associated with the boundaries of the QBL–COX SNP desert segment. A high resolution LD map (1 SNP/1.8 kb) covering ~855 kb was constructed based on genotyping data from a panel of 180 CEPH founder chromosomes [5]. The centromeric end of the reduced variability segment perfectly coincides with an LD break observed between *DQB1* and *DQB3*. This LD break corresponds to the recombination hotspot shaping the genealogy of the DR15–DQ6 haplotype mentioned above. Figure 4 shows a view of the LD structure *(D′)* of the region where this hotspot can be identified in context with additional recombination hotspots described in the MHC [53]. After a high LD region covering *DQB1* (~44 kb), LD is interrupted towards the telomeric part of the *DRB1/DQB1* segment involving a region including the *DRB1* and *DRB2* genes which, owing to lack of SNP-typing information, has been designated as a region of depleted SNP data in Figure 4. The lack of SNP-typing information is a result of significant genotyping failure most likely attributable to the highly polymorphic nature of this region. Consequently, the presence of LD breaks (recombination) on this edge of the referred DNA segment cannot be established.

The region showing the next lowest variation between COX and QBL is a 94 kb section of DNA between *TAP1* and *HLA-DMB* in which only 15 SNPs are found and results in a 2-fold higher π value of 1.6 × 10^−4^ although not approaching the π value of the 158 kb segment.

### The RCCX Region

Within the class III region, there is structural variation in DNA sequence resulting in the duplication of a module termed RCCX, which contains the complement component gene, *C4,* along with portions of adjacent pseudogenes [54]. *C4* has been classified into two versions, *C4A* and *C4B* [55]. The two genes show differences in amino acid sequence in their corresponding protein products, which alters their binding properties [56].

The PGF haplotype possesses a bimodular distribution (Chromosome 6 build NCBI35 32056288..32089024, and 32089025..32121879) with two copies of *C4* as *C4B* and *C4A* respectively [18], both of the “long” form which includes a HERVC4 insertion in intron 9. The COX haplotype, which has a monomodular conformation (AL662849.8 66699 to AL662828.5 6726) containing *C4B,* also differs in that the gene is of the “short” form without the HERVC4 insertion. Although QBL has a monomodular conformation (AL844853.23 134656 to AL929593.6 16047) similar to COX, the *C4* gene present is “long”, and a *C4A*.

Comparison of the coding sequences of these four copies of the *C4* gene not only confirms the published [54] differences between *C4A* and *C4B* in exon 26 and exon 28 but also suggests that there are a further two structural variations (at coding bases 2719, exon 21; and 3218 exon 25) (Table S3). A synonymous variation at coding base 2475 (exon 20) appears to be specific to COX *C4B,* whereas nonsynonymous variations at coding bases 3527 and 3856 (exons 28 and 29) appear to be specific to PGF *C4A*.

## Discussion

We have sequenced 4.25 Mb of a common MHC haplotype that is associated with certain autoimmune diseases, including type 1 diabetes. The full variation content has been defined in relation to two complete MHC haplotypes that we have previously sequenced [18]. The cell lines used in this study were invaluable in obtaining homozygous DNA from the classical MHC and allowed the determination of a complete inventory of polymorphism and sequence across the whole MHC in the context of HLA-defined haplotypes, including gene, pseudogene, promoter, intergenic and complex repeat sequences.

Our approach of cloning and shotgun sequencing, instead of direct sequencing of PCR product [57], evaded the potential pitfalls inherently associated with PCR-amplification within the MHC region, such as mispriming in under-characterised highly polymorphic areas. In addition, many sequences are difficult to PCR for more general reasons, for example, the GC-rich 5' UTRs of genes. The validity of our experimental strategy is reflected by the completeness of the polymorphism map, from SNPs to large DIPs. The identification of significantly altered coding sequences in different haplotypes stresses the value of careful and thorough annotation. The results of these efforts will ensure that the MHC research community has comprehensive genomic information for medical research. The study design will serve as a model system for future sequencing projects of other complex, polymorphic immune gene clusters of the human genome that are associated with disease, such as the leukocyte receptor complex (LRC).

HLA haplotypes in QBL and COX cell lines share identical alleles at *HLA-DRB1* (*0301) and *-DQB1* (*0201) genes and, therefore, some commonality in their origin, composition and function. We were able to define this shared portion of DNA identical by descent to only a small 158-kb segment telomeric of *HLA-DRB3* and centromeric of *HLA-DQB3*. When comparing these two cell lines, this segment presents exceptionally low divergence relative to other regions within the MHC. Outside this segment, the divergence between these haplotypes is as extensive as that we found previously between two HLA-disparate haplotypes: We identified about 15,000 SNPs, of which approximately 40% were novel to the newly sequenced haplotype. Approximately 2,000 DIPs were also identified. The nucleotide heterozygosity between the two haplotypes was 3-fold higher than typical genome-wide diversity. In contrast, the extreme conservation of the 158-kb segment points to a relatively recent common ancestor fewer than 3,400 generations ago.

A number of factors contribute to the variation within the MHC and could potentially be responsible for the existence of the shared 158-kb segment, including conventional and gene-conversion–mediated recombination [1,58]. We propose that this segment originated by conventional recombination, possibly involving recombination hotspots 1 and 2 (Figure 4), giving rise to an original region of about 450 kb. This is supported by extremely low sequence divergence (π = 8.47 × 10^−7^) within the 158-kb segment and is continued by lower than expected sequence divergence within the remaining approximately 290 kb up to the recombination hotspot between *NOTCH4* and *C6orf10*. At both ends, the divergence collapses at LD breaks coinciding with confirmed recombination hotspot 2 [52] at the centromeric end and predicted hotspot 1 at the telomeric end. To our knowledge there has never been a gene conversion–mediated recombination event described involving more than 10 kb of sequence. According to the HLA allele frequencies, the MHC can be divided into only a few blocks that contain non-randomly associated alleles at different loci [59]. The HLA-DRB1*0301–DQB1*0201 (DR3*–*DQ2) block is present in a number of populations, including Caucasians (Whites of northern and western European ancestry), ethnic Africans and Filipinos [59–61], and is often associated with type 1 diabetes, coeliac disease, autoimmune thyroid disease, and multiple sclerosis incidence [25,60,62]. This shared DR3*–*DQ2 identical-by-descent segment or “frozen block” [63] is the most commonly observed DR/DQ haplotype in different European populations in which the ancestral MHC haplotypes A1-B8-Cw7-DR3-DQ2 (e.g., COX) and A30-B18-Cw7-DR3-DQ2 account for by far the largest proportion of its frequency. These extended haplotypes are generally believed to have arisen from their rapid expansion across Europe driven by the selection pressure for the function of a single locus or multiple functional loci of the haplotypes [64]. However, DR3*–*DQ2 has also been observed constituting other much less frequent extended haplotypes [65–67]. The wide distribution of the conserved block in Old World haplotypes deserves further investigation. Because this segment has not been split by recent recombination events, the small number of minor variants distributed over it presumably occurred by mutation. By scoring them in DR3*–*DQ2 blocks in different populations, we will be able to track an accurate clade structure that can be used for timing of association with different flanking regions in relation to population structure and disease association.

**Figure 5:**
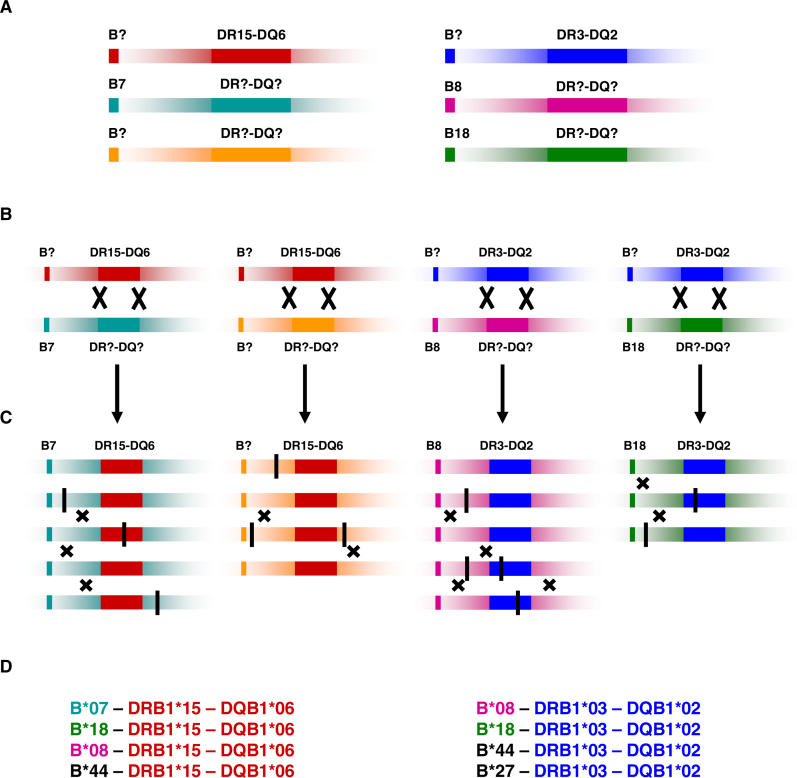
Model of Haplotype Divergence over DR–DQ Region in Relation to Extended MHC Haplotypes (A) Divergence of DR–DQ region over tens of millions of years [73]. (B) Transfer of divergent blocks into other haplotypes by recombination. This does not need a double crossover but could occur by single crossovers separated in time. (C) Relative expansion of ancestral DR–DQ haplotypic segments that may result from either positive selection or neutral processes. Black vertical stripes represent occasional SNP mutations occurring within the MHC including within the ancestral haplotypic segments. Small crosses represent crossovers occurring more frequently outside the conserved DR–DQ blocks relative to inside these blocks. However, allele or gene conversion may take place by closely spaced double crossovers, resulting in diversification of the peptide-binding groove without flanking recombination [87,88]. This model was predicted by Gaudieri et al. (1997) [63] based on incomplete sequence analysis. (D) Examples of contemporary MHC haplotypes containing ancestral DR–DQ segments (data provided by www.allelefrequencies.net).

Our model is, therefore, consistent with the idea that a DNA segment derived from an ancestral haplotype has been transferred into a number of diverse and widely distributed haplotypes by recombination [63,68], and that certain recombinant haplotypes have subsequently expanded in frequency across European populations (see Figure 5). The data suggest that ancestral DR–DQ blocks have been shuffled into different MHC haplotypes. The expansion of the resultant novel haplotypes could relate either to selection for resistance to disease by offering an evolutionary advantage in terms of HLA class II functions and peptide binding specificities, for example, or to neutral genetic drift, perhaps in an ancestral population with a small effective population size. Although not proven, recent data support the long-held view that sequence variation within HLA genes is driven by resistance to infection [69,70]. The spread of the DR3–DQ2 ancestral segment by inter-haplotype exchange may also have been driven by selection. This interesting hypothesis might be tested in further studies by, for example, haplotype-based tests for positive selection [71]. It is not trivial, however, to explain the contrasting genealogy of ancestral haplotype segments in a chromosome. If ancestral DR/DQ haplotypes (i.e., DR3–DQ2) have exchanged the discrete segment of the MHC that appears identical by descent between COX and QBL so recently, it might be possible that similar exchange of different HLA sequences between haplotypes of this defined DR/DQ segment may be responsible for contrasting disease risk related to non-DR/DQ loci [25] and to different ethnic backgrounds [72]. An alternative explanation would require the action of purifying selection on this fragment keeping substitution rates low. However, this argument requires the majority of the DNA to be functional and therefore intolerant of substitutions. The differential contributions of selection and recombination in shaping the contrasting evolutionary history of ancestral haplotype segments containing classical HLA class II genes might be categorized in further studies expanding the population range and increasing SNP density.

The “modular” or block structure of the MHC is well known to the HLA community [59,63,68]. Whether the maintenance of polymorphic conserved and common blocks such as the *DR*–*DQ* segment is due to suppression of recombination or selection has never been satisfactorily resolved [73]. It has been argued that it is advantageous to maintain clusters of polymorphic genes whose products interact [1]. The *DQA* and *DQB* loci are good examples because these polymorphic genes encode a heterodimeric molecule with constraints on pairing of the α and β protein chains [74]. Similarly, different *DR*/*DQ* allelic pairs could be advantageous since they perform interrelated functions. These considerations may lie behind the characteristics of the MHC of ancient, highly diverged haplotypes that appear to be evolving independently except for sequence in the peptide-binding grooves and rare “block shuffling” as we indicate here. They also lie behind the difficulties in locating single gene contributions to disease in which multiple linked interacting genes are at work [25,75]. Our results point to a particular selective advantage of the 158-kb–segment allelic variation in the history of Europeans.

The data generated from the MHC haplotype project provide a major resource for the construction of informative and high-resolution genetic maps in a region that has been more refractory to certain whole-genome analysis methods than less complex regions of the genome. Characterisation of fine segmental LD structure is an essential part of disease mapping, because it provides guidance for the selection of markers [76]. To date, over 40,000 variations from the project have been submitted to dbSNP. Over 60% of the mapped variations in this region were novel submissions from this study. These maps will provide a guide to the fine-scale patterns of LD and recombination within the MHC and will aid methods used to identify optimal sets of tag SNPs that allow association studies to be conducted more efficiently [77]. These methods take advantage of high-resolution maps and can show increasing efficiency at higher marker density [78]. Eventual elucidation of the specific disease-predisposing variants will require detailed association analysis of all genetic differences in tag SNP–defined intervals in a large number of affected individuals and controls, along with functional analysis of associated variants, to verify a biological function consistent with the disease phenotype. The annotation of disease-associated MHC haplotypes in the context of complete information, encompassing all described splice variants of expressed genes and UTR sequences, will provide an initial basis for the subsequent experimental verification of candidate MHC loci and structural variants in disease and in gene expression analyses. The sequences of the remaining haplotypes will not only reveal further polymorphisms for genetic dissection of the MHC in disease, but also define the genealogical relationships between haplotypes. There appears to be differential associations with some immune-mediated diseases and the two B18-DR3 and B8-DR3 haplotype groups studied here. Our data indicate that the variability is probably not determined by sequence variation within the class II gene–containing 158-kb chromosome segment.

Taking together our finding of the conserved sequence block between the DR3-DQ2 COX and QBL sequences, and our observations of a similar level of sequence conservation in the DR–DQ region for DR15–DQ6 haplotypes, a recent inter-haplotype exchange of this discrete portion of the MHC is suggested. The DR–DQ segment is one of the most variable in the genome, yet it is apparently “fixed” in some haplotypes. The precise explanation for this interesting situation needs further investigation, particularly the relative contributions of recombination suppression, selection, and population expansion.

## Materials and Methods

### Cell lines.

The HLA-homozygous typing cell lines, QBL (DR3, Caucasian, Netherlands), COX (DR3, Caucasian, South Africa) and PGF (DR15, Caucasian, England) were selected from the 10th International Histocompatability Workshop panel [19]. The DNA-typing profiles from these cell lines can be found at http://www.ebi.ac.uk/imgt/hla. Following cultivation, DNA from these cell lines was typed to confirm identity and homozygosity (http://www.sanger.ac.uk/HGP/Chr6/MHC).

### BAC clone library construction.

The approach used to construct and screen the PGF and COX BAC libraries, as well as library designations, has been described previously [18]. The QBL library was constructed by Eae-I partial digestion of high-molecular weight DNA, ligation to a 100-fold molar excess of Eae-I to Bst-XI linker-adaptors (which change all EaeI cut “sticky ends” into 3′ overhangs with the sequence CACA), and sorting for high molecular weight DNA by three consecutive passages over 0.5% agarose/TAE gels (gel dimensions 6.4 × 10.1 × 1.0 cm) run in a custom-made orthogonal pulse field mini-gel apparatus. The gel box was made from a Rubbermaid servin' saver 1.2 l plastic container (approx. 16 × 16 × 7 cm), which was raised 1.4 cm off the bench via four plastic “feet” (caps from 15-ml tubes attached with silicon), so that air blown from a small fan could continuously cool the undersurface of the apparatus. The alternating electric fields (45° to the right of direction of DNA travel, alternating with 45° left) were supplied by four 11.5 cm–long platinum electrodes mounted inside, along the base of each of the four sides of the container. Gels were run for 12 h at 70 V, with switch times varying uniformly from 2–14 s over the course of the run.

After each pulse-field run the desired part of the gel (DNA ≥ 120 kb) was excised and DNA recovered by electroelution within dialysis tubing; after the final electroelution, the resulting genomic DNA was ligated into *Bst-*XI + *Sfi-*I doubly-cut vector DNA. The vector used (pDNA-Arts.BAC1) is derived from pBeloBAC11; when cut with BstXI and SfiI a short insert containing the pUC18 origin of replication is liberated from within the polylinker, and the two vector ends are left having identical 3′ overhangs: TGTG. Prior to ligation with genomic DNA, the vector was separated from the short pUC origin insert by gel exclusion chromatography over sephacryl S1000 superfine (column dimensions 26 × 0.4 cm), and aliquots of prepared vector and size-selected genomic DNA were analyzed on 0.5% agarose/TAE gels in order to estimate how much of each DNA preparation to use in the ligation reaction (target ratio for genomic DNA mass:vector DNA mass was 20:1).

Ligation reactions were transformed into Escherichia coli strain DH10B via electroporation using frozen electro-competent cells purchased from Invitrogen (Carlsbad, California, United States). In total, 357 individual 384-well plates were robotically arrayed (137,088 individual clones) to yield the library designated “DAQB.” The library was gridded onto 17 separate 22 × 22 cm nylon filters (Genetix, New Milton, Hampshire, United Kingdom) and screened with a mixture of 164 different ^32^P-labelled overgo probes, which collectively span the entire MHC region. All positive clones were placed in a new array, which was used to generate 164 identical filters that were then probed with each of the individual overgo probes. This allowed placement of clones in rough order, and a tiling path was determined by a combination of BAC end sequencing and BAC fingerprinting.

BAC clones from CHORI-501 (PGF) and CHORI-502 (COX) libraries can be requested from BACPAC resources (http://www.chori.org/bacpac). BAC clones from the QBL library can be requested from john.elliott@ualberta.ca.

### Mapping, sequencing, gene annotation, and variation analysis.

Methods used for mapping, sequencing, gene annotation and variation analysis have been described previously [18]. For the variation analysis, all splice variants of each locus were included. Of several alignment procedures tested, cross-match gave the most accurate detection of haplotype variations (see [18]), and was, therefore, used for the SNP and DIP analysis in this study. We refer to haplotype differences (polymorphisms) without regard to function or frequency and purely as sequence differences. The ratio of non-synonymous (amino acid–altering) to synonymous (silent) substitutions (N:S) was used as an indicator of selection acting on genes, because synonymous alterations are unlikely to exert a selective advantage.

As previously applied in the comparison of the PGF and COX haplotypes [18], a test was conducted to ensure that QBL BACs were derived from a single haplotype by comparing adequately overlapping reads from the shotgun sequencing strategy extending into adjacent BAC clones.

A gap within the QBL HLA-DR region resulting from a clone deficit was closed by sequencing three LR-PCR products that spanned the approximately 20-kb gap. The LR-PCR primers were as follows: QBL-A sense 5′GTG AGG AGT GAT GGG TGA GA3′ with QBL-A antisense 5′GGA AAT AAG GAG GAG GGA AGG3′, QBL-B sense 5′TAC ATG GGT GTC CTT TCA GC3′ with QBL-B antisense 5′TCC TGG TCT CGC TCT TCT TC3′, QBL-C sense 5′TGG GCA AAA TCT TAC CAA CC3′ with QBL-C antisense 5′TCC TTG GGG CTC AGT TAG TG3′.

All sequences presented in this paper have been submitted to the EMBL/Genbank/DDBJ databases and allocated accession numbers (Table S4). For purposes of clarity, all BAC clones are referred to using their accession numbers. All variations from the study were submitted to dbSNP (http://www.ncbi.nlm.nih.gov/SNP) using the submitter handle SI_MHC_SNP. The annotated haplotype sequences can be accessed using the VEGA database (http://vega.sanger.ac.uk; [33]). The SNP data can viewed in the context of the relevant genome annotation through the GLOVAR genome browser (http://www.glovar.org), and in the context of genetic findings in type 1 diabetes in T1DBase (http://www.t1dbase.org).

### Estimation of LD and divergence.

Genotyping data from 180 CEPH founder chromosomes [5] were used to estimate the strength of LD in an approximately 855-kb DNA segment within the MHC bounded by dbSNP rs2849013 (NCBI35 Chr6; 32,240,568) and rs7754316 (NCBI35 Chr6; 33,095,976). This fragment contains the *DRB/DQB* segment shared by QBL and COX cell lines and neighbouring regions extending over each side where recombination hotspots have been previously mapped. Estimates of pairwise-disequilibrium coefficient—D′ [79]—between SNPs were obtained employing the ldmax program in the GOLD package [80]. Long-range LD patterns—high LD and LD breaks—were visualised using GOLDsurfer program [81]. Detailed haplotype-block structure according to Gabriel et al. (2002) [82] criteria was also derived [83] in order to cross-validate interpretations of the LD landscape in the region investigated and correlate LD breaks with recombination hotspot data available from previous studies [5,51–53].

Sub-samples of 140 CEPH founder chromosomes with available HLA-typing data and phase-known SNP-typing information [5] were selected according to their HLA haplotypes to assess variation level at different MHC loci. The mean number of pairwise differences between all pairs of haplotypes in the sample (Arlequin software, [84]) was used as an estimate of relative divergence for each DNA segment in a sample of chromosomes sharing the DR3-DQ2 haplotypes (*n*=16) common to QBL and COX cell lines. Variation levels were compared between DNA segments extending over *HLA-DRB1* / *HLA-DQB1* genes (158kb), adjacent 160kb on its centromeric side, *HLA-A* (201kb) and *HLA-C* / *HLA-*B genes (201kb). Variation level in these segments was also contrasted in a sample of chromosomes homozygous for the most frequent HLA class II haplotype (DR15–DQ6, *n* = 26) and in another set homozygous for the most common *HLA-C-B* haplotype (HLA-C*0702–HLA-B*0702, n=22).

### Dating of the ancestry of the shared DNA segment.

The number of mutations (N) expected between two contemporaneous sequences of a given length (L) is determined by the mutation rate per nucleotide per year (μ) and the length of time since they last shared a common ancestor (t). Mutations accumulate independently on the two lineages from the common ancestor and so the amount of evolutionary time separating the two sequences is twice the age of the common ancestor, hence:

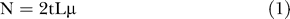



In this scenario, we assumed that the mutation rate was the same on the two lineages. We took two estimates of base substitution rate (μ) from Nachman and Crowell [50], who compared human and chimpanzee divergence. These estimates represent reasonable bounds on the true mutation rate, given the knowledge of the likely speciation time of human and chimps (~6 million years ago) and conservative estimates of the ancestral population size of the common ancestor (between 10,000 and 100,000; [85]). These bounds are 2.1 × 10^−8^ and 1.3 × 10^−8^ per nucleotide per generation.

## Supporting Information

Figure S1 Frequency Distribution of DIP Sizes between Haplotypes (48 KB PPT)

Figure S2 Alignment of Sequences Showing Differing PolyT Tracts from a Single PCR Product (130 KB JPG)

Table S1 Allele Designation of MHC Loci within MHC Haplotypes (45 KB DOC)

Table S2 Genomic Locations of Major DIPs between PGF and QBL (70 KB DOC)

Table S3 Comparison of the CDSs of Four Copies of the *C4* Gene. (53 KB DOC)

Table S4 List of Accession Numbers for QBL Clones (39 KB DOC)

## Abbreviations

BAC: bacterial artificial chromosome
bp: base pairs
CDS: coding sequence
CEPH: Centre d'Etude du Polymorphisme Humain
dbSNP: Single Nucleotide Polymorphism database
DIP: deletion/insertion polymorphism
HLA: human leukocyte antigen
LD: linkage disequilibrium
MHC: major histocompatibility complex
SNP: single nucleotide polymorphism
VEGA: Vertebrate Genome Annotation
