# Supplementary material for: Genetic Analysis of Completely Sequenced Disease-Associated MHC Haplotypes Identifies Shuffling of Segments in Recent Human History
Source: PLoS Genet. 2006 Jan 27;2(1):e9. doi: 10.1371/journal.pgen.0020009 (PMC1331980; doi:10.1371/journal.pgen.0020009)
Supplement: Supplementary file 1 [file pgen.0020009.st001.doc]

**Table S1**

## Allele designation of MHC loci within MHC haplotypes

|  | **PGF** | **COX** | **QBL** |
| --- | --- | --- | --- |
| **A*** | A*030101 | A*010101 | A*2601 |
| **B*** | B*070201 | B*0801 | B*180101 |
| **Cw*** | Cw*070201 | Cw*070101 | Cw*050101 |
| **E*** | E*010302 | E*0101 | E*0101 |
| **F*** | F*010101 *** | F*010101 | F*010101 |
| **G*** | G*010101 † | G*010102 † | G*010102 † |
| **MICA*** | MICA*00801/2 | MICA*00801 | MICA*001 |
| **MICB*** | MICB*004† | MICB*008 † | MICB*00502 † |
| **DRA*** | DRA*010202 ** | DRA*010202 | DRA*0101 |
| **DRB1*** | DRB1*15011 | DRB1*030101 | DRB1*030101 |
| **DRB2*** | absent | novel | novel |
| **DRB3*** | absent | DRB3*01010201/2 | DRB3*020201 |
| **DRB4*** | absent | absent | absent |
| **DRB5*** | DRB5*010101 | absent | absent |
| **DRB6*** | DRB6*0201 | absent | absent |
| **DRB7*** | absent | absent | absent |
| **DRB8*** | absent | absent | absent |
| **DRB9*** | DRB9*0101 † ‡ | DRB9*0101 † ‡ | DRB9*0101 † |
| **DQA1*** | DQA1*010201 | DQA1*050101 | DQA1*050101 |
| **DQB1*** | DQB1*0602 | DQB1*020101 | DQB1*020101 |
| **DPA1*** | DPA1*010301 | DPA1*010301 | DPA1*010301 |
| **DPB1*** | DPB1*040101 | DPB1*030101 | DPB1*0202 |
| **DMA*** | DMA*0101 | DMA*0101 | DMA*0101 |
| **DMB*** | DMB*0101 | DMB*0103 | DMB*0101 |
| **DOA*** | DOA*010102 | DOA*010105 | DOA*01010201/2 |
| **DOB*** | DOB*010101 | CX cds is novel | sequence gap |
| **TAP1*** | TAP1*0101 † | TAP1*0101 † | TAP1*0101 † |
| **TAP2*** | TAP2*0101 | CX cds is novel | QB cds is novel |

†	CDS is extended in the MHC halpotypes with respect to the allele reference sequence (http://www.anthonynolan.org.uk/HIG/data.html)

‡	99% match

**	with 1 snp G/A at base 354

***	with 1 snp T/C at base 814
