# Supplementary material for: Genetic Analysis of Completely Sequenced Disease-Associated MHC Haplotypes Identifies Shuffling of Segments in Recent Human History
Source: PLoS Genet. 2006 Jan 27;2(1):e9. doi: 10.1371/journal.pgen.0020009 (PMC1331980; doi:10.1371/journal.pgen.0020009)
Supplement: Supplementary file 2 [file pgen.0020009.st002.doc]

**Table S2**	Genomic locations of major DIPs between PGF and QBL

| **appox. start position**  **in Chr 6 NCBI35** | **Length**  **(bp)** | **Repeat Element** | **present in haplotype** | | | **context** |
| --- | --- | --- | --- | --- | --- | --- |
|  |  |  | **PGF** | **COX** | **QBL** |  |
| 29788432 | 140 | AluYa5/8 partial | N | N | Y | within HCP5P15 |
| 29793466 | 2593 | 12.2 copies 19 mer (CTCCCTCTCCCGTCTCCCT) & SVA | Y | N | N | between HCP5P15 and HLA-F |
| 29871277 | 209 | 4 mer (TCTT); 59 copies in PGF; 6.8 copies of (TTTA) in QBL and COX | Y | D | D | between HCP5P15 and HLA-F within DAQB-335A13.14 Transcript |
| 29951921 | 5157 | HERVK9 in MER9 | Y | N | N | between MICF and HLA-H |
| 30007761 | 1745 | 13.8 copies 6 mer (CCCTCT) & SVA | Y | N | N | between HLA-K and HLA-21 |
| 30076710 | 122 | 30.8 copies 4 mer (AAGG) | N | N | Y | between HCG9 and HCP5P2 |
| 31079559 | 276 | AluYb8 (within AluSx) | N | N | Y | between C6orf205 and HCG22 |
| 31116369 | 4718 | Complex (3 events): |  |  |  | between C6orf205 and HCG22 |
|  |  | Part of ERVL element (215 nt) & part of MER63 (361nt) | Y | N | N |  |
|  |  | AluSx, MIR, AluJb | N | Y | Y |  |
|  |  | Mer63 & AluY inverted (with respect to PGF) | D | S | S |  |
| 31302355 | 848 | AluY inserted in LTR39-int (HERV3-like) region in PGF & COX | Y | Y | N | between HCG27 and HLA-C |
| 31319617 | 1487 | SVA & GC-rich | Y | N | N | between HCG27 and HLA-C |
| 31358206 | 281 | AluY partial into L1MDa | N | N | Y | between RPL3P2 and WASf5P |
| 31465639 | 96 | 47.5 copies (TC) in PGF & COX; 15.5 copies (TC) in QBL | S | S | D | between HLA-S and MICA |
| 31484791 | 102 | L1MB3 longer in QBL (than PGF & COX) inc. 26 copies of TG 2mer | S | S | D | within MICA |
| 32326029 | 170 | 85 copies 2 mer (TA) in QBL | N | N | Y | between NOTCH4 and C6orf10 |
| 32421759 | 309 | AluYb8 (1-310) into L2 | Y | N | N | within C6orf10 |
| 32648132 | 112 | 2.7 copies 41 mer in PGF | Y | N | N | 6kb telomeric of HLA-DRB1 3' end |
| 32655577 | 454 | AluYa5 within LTR12 in PGF | Y | N | N | within HLA-DRB1 |
| 32660730 | 450 | Longer L2 (QBL/COX: 579nt; PGF129nt) & MER20 partial (165 nt) in QBL/COX | N | Y | Y | within HLA-DRB1 |
| 32663138 | 613 | AluSq & AluY within L2 | N | Y | Y | within HLA-DRB1 |
| 32669946 | 5119 | AT rich /SVA | Y | N | N | between HLA-DRB1 and HLA-DQA1 |
| 32679328 | 270 | AluY | Y | N | N | between HLA-DRB1 and HLA-DQA1 |
| 32700406 | 106 | L1PA2 | Y | N | N | between HLA-DRB1 and HLA-DQA1 |
| 32701690 | 304 | AluY in MER2B | Y | Y | N | between HLA-DRB1 and HLA-DQA1 |
| 32702457 | 155 | L1M4 into MIR | N | Y | Y | between HLA-DRB1 and HLA-DQA1 |
| 32721422 | 653 | L1PA4 (partial) & simple repeat | N | Y | Y | between HLA-DQA1 and HLA_DQB1 |
| 32722941 | 325 | AluSx in L1MC5_Hs | Y | N | N | between HLA-DQA1 and HLA_DQB1 |
| 32724029 | 4074 | L1PA2, HAL1, AluY & AluYd2 | Y | N | N | between HLA-DQA1 and HLA_DQB1 |
| 32729196 | 794 | SVA (partial) & T rich | N | Y | Y | between HLA-DQA1 and HLA_DQB1 |
| 32730492 | 315 | AluSx | Y | N | N | between HLA-DQA1 and HLA_DQB1 |
| 32731502 | 2335 | MER11C & LTR5 | Y | N | N | between HLA-DQA1 and HLA_DQB1 |
| 32739664 | 311 | AluY | N | Y | Y | within HLA-DQB1 |
| 33134701 | 2223 | SVA partial (length COX<PGF<QBL) | D | D | D | between HLA-DOA and HLA-DPA1 |
| 33201403 | 315 | AluYb8 | Y | Y | N | within HLA-DPB2 |

S = same, D= different, Y = present, N = absent
