# Supplementary material for: Genetic Analysis of Completely Sequenced Disease-Associated MHC Haplotypes Identifies Shuffling of Segments in Recent Human History
Source: PLoS Genet. 2006 Jan 27;2(1):e9. doi: 10.1371/journal.pgen.0020009 (PMC1331980; doi:10.1371/journal.pgen.0020009)
Supplement: Supplementary file 3 [file pgen.0020009.st003.doc]

**Table S3**

Comparison of the coding sequences of four copies of the *C4* gene

| **Coding sequence base** | ***C4A* PGF** | ***C4A* QBL** | ***C4B* PGF** | ***C4B* COX** | **Exon** | **Amino acid change if any** | **Status** |
| --- | --- | --- | --- | --- | --- | --- | --- |
| 2475 | C | C | C | T | 20 | V825V | s |
| 2719 | G | G | A | A | 21 | A907T | n |
| 3218 | A | A | G | G | 25 | D1073G | n |
| 3359 | C | C | T | T | 26 | P1120L | k |
| 3362 | G | G | C | C | 26 | C1121S | k |
| 3370 | T | T | A | A | 26 | I1124L | k |
| 3373 | G | G | C | C | 26 | D1125H | k |
| 3375 | C | C | T | T | 26 |  | k |
| 3527 | A | G | G | G | 28 | N1176S | s |
| 3615 | G | G | C | C | 28 | A1205A | k |
| 3620 | T | T | C | C | 28 | V1207A | k |
| 3629 | T | T | G | G | 28 | L1210R | k |
| 3630 | C | C | G | G | 28 |  | k |
| 3856 | T | G | G | G | 29 | S1286A | s |

Coding sequence base refers to the position in the CDS where the base A of the ATG initiator Met codon is denoted nucleotide +1.

s = possible real SNP, n = novel C4 variation, k = known C4 variation
