# Supplementary material for: Genetic Analysis of Completely Sequenced Disease-Associated MHC Haplotypes Identifies Shuffling of Segments in Recent Human History
Source: PLoS Genet. 2006 Jan 27;2(1):e9. doi: 10.1371/journal.pgen.0020009 (PMC1331980; doi:10.1371/journal.pgen.0020009)
Supplement: Supplementary file 4 [file pgen.0020009.st004.doc]

**Table S4**

List of accession numbers for the QBL clones

Clone Name	Accession #

DAQB-239N17	AL844539

DAQB-5M14	BX000360

DAQB-73K12	BX537153

DAQB-314F7	AL954865

DAQB-117O11	AL929561

DAQB-153A4	AL935156

DAQB-169K4	AL929573

DAQB-283L15	BX296534

DAQB-304F3	BX004810

DAQB-12N14	BX000531

DAQB-36F16	BX000688

DAQB-92E24	AL929591

DAQB-335A13	AL844851

DAQB-346J13	BX001005

DAQB-90C11	AL845454

DAQB-215D11	AL845439

DAQB-112K1	BX322644

DAQB-44E15	AL844220

DAQB-256M13	AL845452

DAQB-170I5	AL773535

DAQB-39G10	AL844203

DAQB-352F19	AL844213

DAQB-256B5	BX000357

DAQB-298I5	BX248507

DAQB-47P19	AL845353

DAQB-230F21	AL773551

DAQB-10J12	AL805917

DAQB-196C18	AL773589

DAQB-11J22	AL773541

DAQB-218D23	AL805909

DAQB-185I1	AL773544

DAQB-5G13	AL845458

DAQB-115I13	BX322539

DAQB-48K1	AL845443

DAQB-72H16	BX001040

DAQB-87N14	AL929587

DAQB-195H10	AL805934

DAQB-350E15	AL844216

DAQB-147D11	AL929592

DAQB-331I12	AL844853

DAQB-124C11	AL929593

DAQB-143M3	AL845464

DAQB-282H15	AL845509

DAQB-309N16	BX001039

DAQB-184K4	AL935032

DAQB-97F8	AL929581

XX-BQB97F8_109B10_3	CR854861

XX-BQB97F8_109B10_2	CR854860

XX-BQB97F8_109B10_1	CR854859

DAQB-109B10	AL935026

DAQB-187P15	AL773543

DAQB-69D7	AL935043

DAQB-96B10	AL935042

DAQB-22D21	AL805913

DAQB-79P13	AL845446

DAQB-314F24	AL844527

DAQB-193B15	BX000343

DAQB-126H3	BX248088
